# The Influence of Knowledge Management Capacities on Pharmaceutical Firms Competitive Advantage: The Mediating Role of Supply Chain Agility and Moderating Role of Inter Functional Integration

**DOI:** 10.3389/fpubh.2022.953478

**Published:** 2022-07-05

**Authors:** Zhihua Hu, Muddassar Sarfraz, Kausar Fiaz Khawaja, Hina Shaheen, Shahida Mariam

**Affiliations:** ^1^Department of Computer Science, Huanggang Normal University, Huanggang, China; ^2^School of Management, Zhejiang Shuren University, Hangzhou, China; ^3^Faculty of Management Sciences, International Islamic University, Islamabad, Pakistan

**Keywords:** absorptive capacity, transformative capacity, inventive capacity, supply chain agility, inter-functional integration, competitive advantage, resource-based view theory

## Abstract

This study investigates the factors such as knowledge management capacities and their positive influence on firm competitive advantage or the supply chain agility of the firm and the underlying mechanisms (supply chain agility) that facilitate the firm's performance and leads to firm competitive advantage. It also explores the moderating role of inter-functional integration. We have collected the data from the 308 supply chain managers of pharmaceutical firms in Pakistan and questionnaires were used for data collection with multi-item scales already developed and validated. The findings suggest that knowledge management capacities significantly influence a firm's competitive advantage or supply chain agility. The supply chain agility fully mediates between absorptive capacity, transformative capacity, and firm competitive advantage. Further, supply chain agility partially mediates between inventive capacity and firm competitive advantage. Meanwhile, inter-functional integration moderates the relationship between supply chain agility and firm competitive advantage, with their positive relationship strengthening when inter-functional integration is high. The study provides empirical evidence that knowledge management capacities (such as absorptive capacity, transformative capacity, and inventive capacity), supply chain agility, and inter-functional can be important factors in improving firm performance.

## Introduction

Over the years, competitive knowledge in the new economy has altered the business landscape, thus becoming a critical ingredient for gaining advantage. The increase in globalization and competition has lead global industries to utilize knowledge as a strategic business asset to foster firms' competitiveness. In today's fast-growing environment, knowledge plays a fundamental role in firms' competitive development. Knowledge is a valuable resource that helps a business quickly respond to market changes, gaining a substantial competitive advantage (CA) as a result (1).

In particular, firms perform better than competitors on the basis of valuable knowledge. In recent decades, knowledge has become a valuable asset for firms on the basis that a firm can survive and target their competitors and achieve CA (2). However, in such a competitive time, it is noteworthy that knowledge works as a competitive weapon, managing business uncertainty.

A firm needs knowledge that helps them to respond quickly to market changes and adopt changes frequently at the right time. Changing market trends allow firms to pay greater attention to KM (3). KM is a set of dynamic capabilities of a firm to gather valuable information and then send it across the units of the firm to improve operations internally (4). These dynamic capabilities allow a firm to update and redesign their process with changing market trends. KM is the precise treatment of learning and potential information (5). It is important since it surveys the company's capacity to recognize, absorb, change, and apply profitable external as well as internal information to their procedures or activities to gain an upper hand.

KM is essential because of the growing need to develop a competitiveness capacity to compete in a world economy. Accordingly, today, business landscapes have experienced vast and rapid changing market trends, causing numerous organizations to invest in KM practices. The turbulent market uncertainties have elevated the need for developing novel KM capabilities to combat the emerging business challenges. Realizing the underlying significance of a firm's KM capability has created a reflex in the organizational processes whereby information and knowledge have fundamentally become the catalysts for the development of CA. Given this explanation, the literature indicates that knowledge assets create value for the firm, thereby becoming the prime determinant of superior CA (6).

With the increasing research in the KM domain, supply chain agility (SGA) as a mechanism that explains the relationships between KM capacity and firm outcomes has received more attention, with management adopting KM practices to achieve business competitiveness. In this regard, organizations view gaining a CA as imperative to the operation of the supply chain management process. As such, SGA has emerged as a strategic capability for improving firms' performance and competitiveness (7). Effective supply chain orientation provides distinctive CA to firms. In explaining this notion, the research states that it enables organizations to meet market demands by effectively synthesizing firms' capabilities and competitiveness (8). Undoubtedly, knowledge plays an important role as it is an intangible resource for a firm. It is believed that knowledge management capacities may help a company to enhance the agility of its supply chain and due to SCA, a firm may gain CA. While numerous studies address SGA but very few studies have examined it as mediator, and there is little research on the factors that impact SCA. In addition, there is still a lack of studies in the context of KM and its influence on SCA (9).

Therefore, the present study aims to expand the existing KM literature by investigating the KM capacities of firms, such as their Absorptive capacity (AC), Transformative capacity (TRC), and Inventive capacity (INVC), and their influence on firm CA. The purpose of the research is to identify the influence of KM capacities on firm CA. The research also investigates the mechanism of SCA through which the enterprise gains a CA. Furthermore, the study considers the role of inter-functional integration (INTF) as a moderator between SGA and the CA of the firm.

Significantly, this study makes an important theoretical contribution to the role of the SCA of the firm. This study is unique in exploring several aspects. It considers how KM capacities affect a firm's CA and also how KM capacities impact SCA. The previous research examines the mediating impact of SCA between AC and company performance (10). Therefore, this research examines the mediating role of SCA between KM capacities and firm CA for the first time. The current research also examines the moderating role of inter-firm integration between SCA or firm CA for the first time. To date, there is no research on KM capacities and their influence on SCA and firm CA. Therefore, this current study fills a gap in the literature by investigating the INTF between SCA and CA for the first time.

The findings of the current research provide direction to supply chain managers to overcome continuous challenges by applying effective KM. The combination of these three knowledge capacities (AC, TRC, and INVC) will help managers understand which contributes most to their firm's outcomes. Further, the present study will provide direction to managers to retain focus on INTF as it becomes a major issue. It will benefit the company by drawing their attention to this crucial problem and allowing them to understand that overcoming this problem is essential for the company to succeed, by speeding up the SCA and achieving CA.

This study consists of six different sections. The introduction section illustrates the study's purpose, the research objectives, and significance. Section Literature Review demonstrates the hypothesis development, while Section Literature Review outlines the research methodology. Section Results explains the study outcomes, and Section Discussion compares those outputs with the previous literature. Lastly, Section Conclusion concludes the research topic by explaining the study's limitations and implications.

## Literature Review

### Knowledge Management Capacities and Competitive Advantage

An organization can achieve CA by having valuable resources that help in achieving firm performance; these resources can be tangible or intangible (11). In today's global competition, a business survives on not only by having tangible resources but also needs to have intangible ones. The fast-growing nature of modern business competition exists on the basis on intangible resources such as valuable information, i.e., the knowledge that can help in achieve performance quickly. In this manner, a firm with strong KM may respond significantly more successfully to clients' needs with new or adjusted products and compete with their competitors at the right time to gain a powerful position in business (12). Indeed, KM has emerged as a key strategic tool to manage firms' competitive performance. Given the articulation, the study shows that knowledge is a significant factor that encourages the firm to achieve competitive benefit over others (13). Notably, knowledge management is a prime determinant of firms' competitiveness. In explaining this notion, the study states that knowledge management profoundly accelerates the firms' performance, thereby obtaining superior competitive benefits (14).

Global organizations have started developing knowledge as a strategic resource for gaining business competitiveness. In the modern era, this realization has caused organizations to acquire knowledge effectively to attain a superior CA (15). In explaining this notion, the research states that strong market competition has lead organizations to try to gain a CA by improving their KM practices (16). As such, the current study proposes the following hypothesis.

#### Absorptive Capacity and Competitive Advantage

Absorptive capacity (AC) is a firm's ability to explore information externally or to search for information from an external source outside the firm's boundaries (17). In recent years, many organizations have faced undeniable market challenges, thus bolstering the need to maintain a competitive position. In this regard, AC has emerged as a successful innovation, thereby providing competitive benefit. AC helps firms tackle market uncertainties to gain a CA (18). Moreover, the literature states that a company's AC strengthens the firm's knowledge process, substantially providing the firm with a CA (19). Significantly, AC is an effective means of gaining a competitive advantage. The absorptive capacity contributes to the firm's performance, thereby accelerating the firm's market dominance. Accordingly, in further explaining this relation, the research reveals that AC improves organizational capabilities and competitiveness (20). Undoubtedly, the idea of the absorptive capacity popularized by various researchers had realized AC as a fundamental tool fostering the firms' competitiveness. Given the illustration, the study reveals that absorptive capacity promotes the firms' internalization, thus gaining a competitive advantage (21). Hence, the literature states that organizations should develop their AC to achieve CA (22). As such, the current study proposes the following hypothesis:

*H1(a): Absorptive capacity positively influences firm competitive advantage*

#### Transformative Capacity and Competitive Advantage

Transformative capacity (TRC) is the ability of a company to generate knowledge internally or to acquire information from past experiences and skills that the people of the firm possess over time (23). The previous literature found that the practice of KM is the selective application of information from prior decisions or experiences to current and future decision-making procedures (24). Empirical evidence shows increasing openness toward knowledge that is gained from past experiences on which a decision is made to improve firm performance (25). company can attain CA by having prior knowledge, experience, and skills that will add to the knowledge base and help in decision making regarding what is needed for the firm to improve its internal processes, products, and services (26).

However, in the era of the knowledge revolution, the firms' transformative capacities have profoundly contributed to achieving the firms' competitiveness. In recent years, knowledge transfer has elevated the transformation capacities to improve firms' competitiveness. The transformative initiative helps the firms to manage their internal knowledge network by significantly achieving sustainable business competitiveness. Given the articulation, the study states that a firm's transformation capacities foster the firm's knowledge management capabilities, thus attaining a competitive advantage (27).

In particular, TRC is the unique valuable intangible assets of the company that help it to attain sustainable CA. As such, the current study proposes the following hypothesis:

*H1(b): Transformative capacity positively influences firm competitive advantage*

#### Inventive Capacity and Competitive Advantage

Inventive capacity (INVC) is a firm's ability to generate information inside the firm by identifying a particular opportunity (23). Over the years, the notion of the firms' innovative capabilities' has come into the spotlight. In the twenty-first century, the firms' innovation capabilities' have played a dominant role in accelerating the firms' competitiveness. The innovation capabilities manage the firms' products and processes conducive to developing competitive advantage. Accordingly, the study shows that the development of the firms' innovation capacities governs the firms' operations, thereby promoting firms' competitiveness (28). Knowledge INVC facilitates a firm's learning process and business performance. Based on this statement, the research shows that a firm's innovation ability helps to improve its business performance and competitiveness (29). Moreover, the INVC plays a fundamental role in creating higher profits and competitive benefits. A firm's INVC capabilities expand its business opportunities by shaping the firm's business model (30, 31), thus establishing a superior competitive edge (6). Indeed, the literature concludes that the effective development of INVC helps an organization achieve a distinctive edge over its competitors (32). Accordingly, the current study proposes the following hypothesis:

*HI(c): Inventive capacity positively influences firm competitive advantage*

### Supply Chain Agility and Competitive Advantage

Supply chain agility (SCA) is the company quick response to the continuously changing market trends (33). The previous literature found that SCA has appeared as an important source of competitiveness in the current period of company instability (10). In recent years, increased market volatility has caused businesses to enhance their supply chain capability to improve their CA. Hence, in this regard, increased attention is given to SCA to speed up a firm's performance and competitiveness. In explaining this notion, the research states that a firm's SCA redesigns the firm's process by improving its business performance and competitiveness (34). Moreover, a firm's SCA magnifies the market demand and the extent to which the firm can respond to substantially satisfy its customers' needs. Accordingly, the literature states that SCA positively accelerates stakeholder demand, thus increasing the firms' performance and competitiveness (35, 36). Altogether, supply chain agility is vital to achieving a competitive advantage. In this regard, the study suggests that to sustain the knowledge economy, companies should retain their competitiveness *via* supply chain agility (37). As such, the current study proposes the following hypothesis:

*H2: Supply chain agility positively influences firm competitive advantage*

#### Absorptive Capacity and Supply Chain Agility

The past literature proposes that real CA is based on a company's potential to use existing information to produce updated items according to need or demand of customers as time passes (10). Significantly, AC improves a fir's' knowledge beyond its boundaries. AC is most relevant to updating a firm's supply chain knowledge, thus rapidly enhancing and satisfying the stakeholders' demand for critical information. Therefore, to justify this relationship, the research states that AC stimulates, transforms, and recognizes sensitive stakeholder information to improve a firm's performance and SCA (38). It is noteworthy that AC develops a significant relationship with the SCA for increasing the firms' knowledge acquisition. Indeed, absorptive capability is an effective means for driving supply chain agility. The absorptive capacity increases the firm's effectiveness, thus contributing to supply chain agility. Based on this statement, the study states that absorptive capacity improves the firms' performance and agility (39, 40). Hence, as a result, the companies should adopt AC to improve the SCA.

Accordingly, the previous literature confirms that higher AC increases organizational learning, thus facilitating SCA. Hence, based on this statement, the research states that AC improves a firm's performance and agility (40). As such, the current study proposes the following hypothesis:

*H3(a): Absorptive capacity positively influences supply chain agility*

#### Transformative Capacity and Supply Chain Agility

Past studies illustrate that the exercise of KM involves the implementation of information to current and later decision-making activities from prior decisions, experiences, or operations (41). Undoubtedly, growing KM in the supply chain has enhanced firms' agility. As a result, today, firms are profoundly incorporating TRC to improve SCA. In the knowledge economy, the novel transformative paradigms have driven the business processes to accelerate firms' innovative capabilities and SCA. Based on this statement, the research states that TRC synchronizes a firm's processes by fostering SCA (42). Indeed, the application of TRC ensures that a firm achieves SCA. Over the years, the TRCs have brought incremental improvements to firms' innovation processes by radically achieving organizational agility. This notion leads firms to embrace transformative practices to rationalize SCA. As such, the researchers state that the digital technological capacities contribute to a firm's SCA, thereby improving its performance (43, 44). As such, the current study proposes the following hypothesis:

*H3(b): Transformative capacity positively influences supply chain agility*

#### Inventive Capacity and Supply Chain Agility

Innovation is the prime determinant of a firm's supply chain operation that helps it to compete in today's business environment. Companies operating in this context have developed INVCs for accelerating supply chain activities. Undoubtedly, the changes in the business environment have led to firms improving their INVCs to meet the customers' demands, thus improving business performance and SCA (45). The previous literature found that in-house research and development has a significant impact on performance and innovation (10). A firm's innovative infrastructure provides information by allowing digital platforms to influence SCA. In particular, the profound characteristics of digital transformations leverage the firms' capabilities to enhance SCA.

With the rapid development in information and technology, the study shows that firms' innovation capacities have improved supply chain agility. The innovation capabilities develop a seamless supply chain connection, thus leveraging the firms' agility performance (46). In explaining this notion, the research states that INVCs strengthen a firm's ability to improve SCA (47). Indeed, the literature confirms that INVCs enhance SCA. In the explanation, the study shows that a firm's IC accelerates its process agility (48). Consequently, the literature concludes that a firm's INVCs and digital transformations are crucial for adapting SCA. As such, the current study proposes the following hypothesis:

*H3(c): Inventive capacity positively influences supply chain agility*

### The Mediating Role of Supply Chain Agility

SCA has emerged as an important factor and has a significant influence on firm performance (49). The success of creating sustainable CA in supply chain management is highly dependent on knowledge and the extent to which it is effectively managed (50). Gaining AC fundamentally inspires an organization to use its knowledge resources to gain sustainable CA. A firm's knowledge capability makes the organization competitive. Moreover, absorptive capacity improves the firms' knowledge acquisition, thereby translating the firm's performance into a sustainable competitive advantage. Perhaps, to understand this concept, the study states that absorptive capacity accelerates the firms' performance, thus raising the business competitiveness (38).

To explain this notion, the research shows that a firm's knowledge capability (i.e., AC) satisfies the needs of the supply chain, thereby making this strategic resource (e.g., SCA) attain superior CA (10). Therefore, by assessing the role of AC in fostering SCA and firms' competitiveness, it has become imperative for organizations to adopt ACs to gain a CA. To theoretically develop the argument on this perspective, the literature has concluded that CA primarily depends on a company's knowledge resources application, accelerating SCA and CA.

In particular, SCA plays a significant role in anticipating market changes and customer demand. Therefore, to survive in today's turbulent landscape, the emerging digitalization has made SCA crucial to establishing a distinctive CA. TRC holds a prominent position in KM by providing organizations with numerous strategic opportunities to achieve firm CA. In explaining this notion, the research states that a firm's TRCs synchronize the supply chain process, thereby increasing the firm's SCA and CA (51).

Further, knowledge significantly fosters organizational INVC and CA (52). As a result, numerous companies have adopted INVC to explore novel business opportunities. In the supply chain, by responding to the new possibilities, firms have embraced INVCs, fostering SCA to achieve CA (53). In particular, in today's competitive market, organizations have adopted INVC to influence the firm's SCA (47), ultimately enhancing its CA.

Significantly, today's organizations have adopted the agility for establishing dominant market competitiveness. In this regard, innovative capabilities play a critical role in achieving competitive advantage. Therefore, today, INVC are recognized as the most significant driver of SCA and CA. However, developing supply chain agility and innovativeness demands the organizations to effectively manage the innovation capacities, thus achieving a competitive advantage (54). In explaining this notion, the literature states that a firm's INVC enhances its SCA (55), substantially leading to CA. Consequently, due to the increasing significance of INVCs in the supply chain, it is suggested that management should improve their knowledge capabilities to increase the value of SCA and CA. Therefore, based on the gathered data, the current study proposes the following hypothesis:

*H4(a): Supply chain agility mediates the relationship between absorptive capacity and competitive advantage*

*H4(b): Supply chain agility mediates the relationship between transformative capacity and competitive advantage*

*H4(c): Supply chain agility mediates the relationship between inventive capacity and competitive advantage*

### The Moderating Role of Inter Functional Integration

Inter-functional integration (INTF) is a firm's internal functional department's ability to collaborate, coordinate, and communicate information or activities among all the departments of the firm who are working toward the same goal and work jointly to achieve the task as quickly as possible (56). The better a firm's INTF, the quicker it will achieve its business goals or react immediately to clients' needs. As mentioned, SCA is the ability to respond to the new trends of the market quickly and effectively by redesigning the process and operation of the firm according to the needs of market. As such, when a firm has strong INTF, those within the firm will work together to update the internal process effectively or to respond faster than competitors at right time in the market changes by satisfying their customer or retaining their customer. In this way, the firm can gain a good position in the business by reacting to changing demands or targeting their competitors to sustain the firm's CA.

Empirical evidence exists that the units working together like this assists in improving new products and processes development (57) as well as enhancing firm performance (58). A prior study explores how interaction among functional units has an influence on work performance (59). In explaining this notion, the study states that INTF allows firms to improve their supply chain performance (60), thereby achieving a superior CA.

Today, organizations are experiencing market pressure to provide superior supply chain services, thus establishing firms' competitiveness. In this regard, the firms' functional integration has emerged as a significant construct that complements the supply chain agility and competitiveness (61).

As a result, many companies have increasingly enhanced their supply chain activities, thus attaining a competitive advantage. Understanding the impact of INTF, firms have realized the significance of using an integrated system for creating and sharing value among the supply chain network. In particular, such integration models develop a competitive supply chain approach that enables firms to achieve market competitiveness. This integrated process provides high value to firms by improving their agility, abilities, and competitiveness (62). Therefore, the use of an integrated model has become a critical strategy for fostering SCA and competitiveness. As such, the current study proposes the following hypothesis:

*H5: Inter functional integration positively moderate the connection between supply chain agility and firm competitive advantage*.

**Figure 1:**
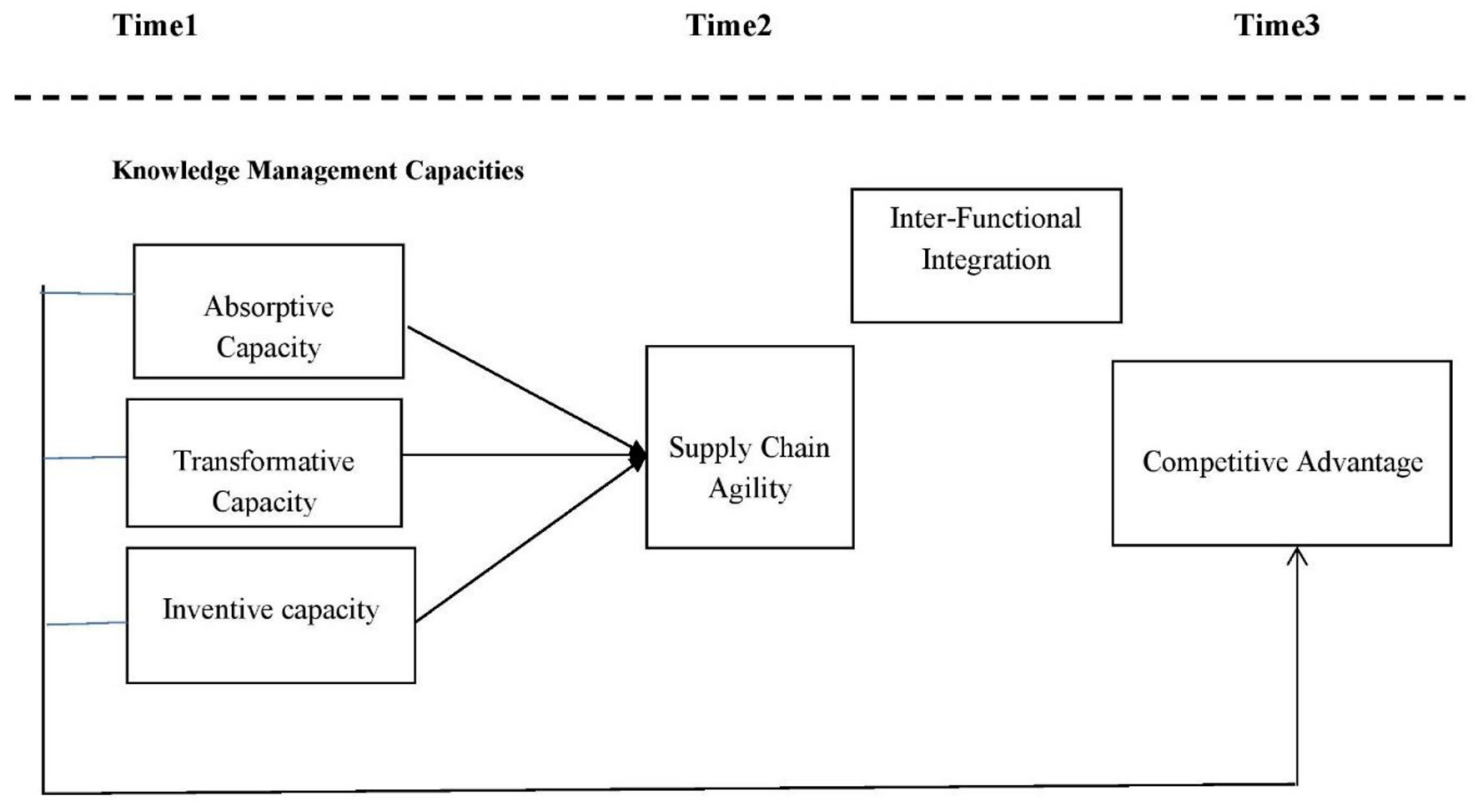
Conceptual framework.

Figure 1 shows study theoretical framework (Independent, dependent, mediating and moderating variables).

## Research Methodology

The purpose of this research study was to investigate the influence of three KM capacities (AC, TRC, and INVC) on firms' CA, the mediating role of SCA between KM capacities, and firm CA, plus the moderating role of INTF between SCA and CA. The supply chain managers of pharmaceutical firms were invited to respond with information about their firm's KM capacity at time 1 and SCA and the INTF at time 2, then firm CA at time 3, and the final sample size was 308 after data screening. Demographic characteristics of the study participants are depicted in Table 1, revealing 89.7% male and 10.3% female. 54.6% participants of age group 31–35; 35.5% were from 36 to 40. 90.2% of participants were master's degree holders, and 9.8% were M.Phil./Others.

**Table 1:** Demographic characteristics.

**Items**	**(%)**
**Gender**
Male	89.7%
Female	10.3%
**Age**
25–30	1.2%
31–35	54.6%
36–40	35.5%
41–50	6.3%
>50	2.4%
**Education**
Master	90.2%
MPhil/Others	9.8%

### Study Measures

Self-administrated questionnaires were developed for data collection. All the questionnaires were distributed in English. In the questionnaire, the first paragraph explained the scope and purpose of the study. Data were collected from supply chain managers of pharmaceutical firms during three different periods. The strict anonymity of the respondents regarding their responses was assured, and their participation was voluntary. The non-probability convenient sampling technique was used for questionnaire distribution. The included items were adapted from established instruments from the previous literature.

Absorptive Capacity was measured using a 13 item scale developed by Jansen et al. (63). Sample items include: “Our unit has frequent interactions with corporate headquarters to acquire new knowledge”; “Our unit regularly considers the consequences of changing market demands regarding new products and services.”

Inventive Capacity was measured using a three item scale developed by Lichtenthaler and Lichtenthaler (23). Sample items include: “Ability to the importance of internally explore knowledge or generate internally new knowledge.”

Transformative Capacity was measured using an eight item scale developed by Lichtenthaler and Lichtenthaler (23). Sample items include: “New opportunities to serve our customers with existing technologies are quickly understood” and “Knowledge management is functioning well in our company.”

Competitive Advantage was measured using a six item scale developed by Vorhies and Morgan (64). Sample items include: “Compared with our competitors, we provide dependable delivery”; “Compared with our competitors, we quickly deliver products to the market.”

The mediating variable supply chain agility was measured using a 22 item scale developed by Garver and Mentzer (65). Sample items include: “Our firm can promptly identify opportunities in its environment”; and “We always receive the information we demand from our suppliers.”

The moderating variable Inter Functional Integration was measured using a 10 item scale developed by Van de Ven and Ferry (66). Sample items include: “To coordinate activities with these other departments during the past 6 months, to what extent have standard operating procedures been established?” It was measured using a five-point Likert scale ranging from “no extent” to “very great extent.” “During the past 6 months, my department are involved with other departments to receive or send work and to receive or send technical assistance.” It was measured using a five-point Likert scale ranging from “not at all” to “very much.”

### Measurement Model Evaluation

Confirmatory factor analysis was used to test the data validity obtained from the Six-factor model. Initially conducted confirmatory factor analysis indicated AGFI, NFI, and CFI values were not in a suggested range (CMIN = 2,040, DF = 766, *p* < 0.000, CMIN/DF = 2.664, CFI = 0.90; GFI = 0.767, RAMSA = 0.071). Therefore, as per the suggestion (67), error terms were connected after observing modification indices values. Hence measurement model was improved with CMIN = 1,792, DF = 764, *p* < 0.000, CMIN/DF = 2.346, CFI = 0.940; GFI = 0.856, RAMSA3 = 0.06. In addition, all items on their corresponding latent variables were positively loaded.

## Results

Illustrates the correlation, reliability, and validity analysis of the study variables (Table 2). The table depicts the analysis values within the range that Hair et al. (67) advised. As shown competitive advantage is significantly related to inventive capacity (*r* = 0.403^**^), transformative capacity (*r* = 0.408^**^) and absorptive capacity (*r* = 0.460^**^). Similarly, inter-functional integration is significantly related to competitive advantage (*r* = 0.079^**^, *p* < 0.05) and supply chain agility (*r* = 0.014^**^).

**Table 2:** Correlation, reliability, and validity statistics.

	**ICR**	**CR**	**AVE**	**INF**	**IC**	**AC**	**SCA**	**CA**	**TC**
**INF**	0.965	0.946	0.745	(0.863)					
**IC**	0.923	0.847	0.734	0.005**	(0.857)				
**AC**	0.905	0.899	0.538	0.032**	0.296**	(0.734)			
**SCA**	0.988	0.968	0.669	0.014**	0.094**	0.154**	(0.818)		
**CA**	0.964	0.964	0.818	0.079**	0.403**	0.460**	0.263**	(0.904)	
**TC**	0.810	0.933	0.778	0.094**	0.662**	0.253**	0.071**	0.408**	(0.882)

**Correlation is significant at the 0.01 level (2-tailed). INF, inter functional integration; IC, inventive capacity; TC, transformative capacity; AC, absorptive capacity; SCA, supply chain agility; CA, competitive advantage; ICR, internal consistency reliability; CR, composite reliability; AVE, average variance extracted; Values on the diagonal represent the square root of the average variance extracted (discriminant validity), while the off diagonals are correlations.

### Hypothesis Testing

Table 3 reveals results of hypothesis H1(a), H1(b), and H1(c); H2, H3(a), H3(b), and H3(c), and H4(a), H4(b), and H4(c) (mediation analysis). Hypothesis 1 states that knowledge management capacity (absorptive, transformative and inventive) is positively related to competitive advantage with β = 0.8678, *p* < 0.001; β = 0.4731, *p* < 0.001; β = 0.6784, *p* < 0.001, respectively; moreover, Hypothesis 2 states that supply chain agility is positively related to competitive advantage with β = 0.343, *p* < 0.001. Hypothesis 3 states that knowledge management capacity (absorptive, transformative and inventive) is positively related to supply chain agility with β = 0.527, *p* < 0.001; β = 0.5093, *p* < 0.001; β = 0.6754, *p* < 0.001, respectively. Hence Hypothesis H1(a), H1(b), H1(c), H2, H3(a), H3(b), and H3(c) are statistically proved. Mediation analysis and indirect effects were calculated using Macro Process by Hayes with Model 4. Preacher and Hayes (68, 69) suggested that if there are no opposite sign in the values of LLCI and ULCI of a relationship; mediation is said to prove. Hence based on the statistics results depicted in with no opposite signs, hypothesis H4(a), H4(b), and H4(c) is proved (Table 3).

**Table 3:** Hypotheses testing.

**Hypothesis**	**Relationships**	**Std. beta**	**Std. error**	***T*** **-values**	***P*** **-values**
H1(a)	AC → CA	0.8672	0.1099	7.8940	***
H1(b)	TC → CA	0.4731	0.0918	5.1547	***
H1(c)	IC → CA	0.6784	0.0512	13.2598	***
H2	SCA → CA	0.343	0.0503	6.814	***
H3(a)	AC → SCA	0.527	0.1168	4.516	***
H3(b)	TC → SCA	0.5093	0.0912	5.5862	***
H3(c)	IC → SCA	0.6754	0.0505	13.3785	0.0000
		**Std. beta**	**Std. error**	**LLCI**	**ULCI**
H4(a)	AC → SCA → CA	0.1809	0.0544	0.0872	0.3004
H4(b)	TC → SCA → CA	0.1900	0.0457	0.1102	0.2902
H4(c)	IC → SCA → CA	0.6836	0.0686	0.5473	0.8153

n = 308. ***p < 0.001; IC, inventive capacity; TC, transformative capacity; AC, absorptive capacity; SCA, supply chain agility; CA, competitive advantage; LL, lower limit; CI, confidence interval; UL, upper limit.

**Figure 2:**
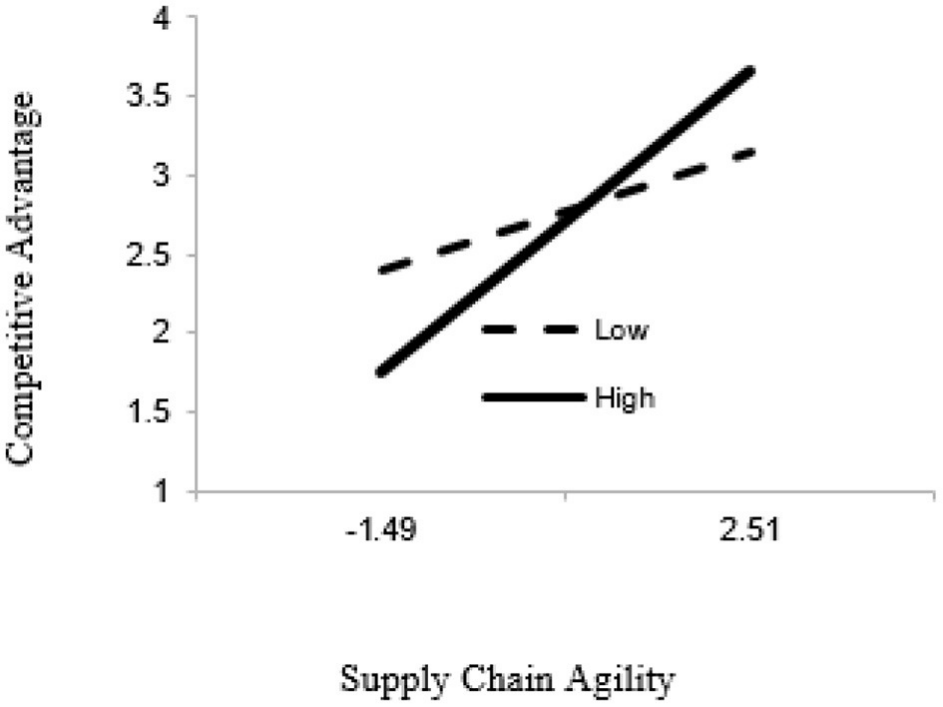
Interaction (SCA*INF → CA).

In line with hypothesis 5, the interaction term of supply chain agility and inter-functional integration was significant (β = 0.2040, *p* < 0.001). Moreover, the results in Table 4 depict that the relationship between supply chain agility and competitive advantage strengthens when inter-functional integration is high (β = 0.4749, *p* < 0.001; see Figure 2).

**Table 4:** Interaction and conditional effects.

**Hypothesis**	**Interaction effects**	**Std. beta**	**Std. error**	***T*** **-value**	***P*** **-value**
**H5**	Interaction SCA*INF → CA	0.2040	0.0725	2.8151	***
	**Level of the moderator**	**Effects**	**Boot SE**	**LLCI**	**ULCI**
**Conditional effects**					
	MOD−1 SD (−0.69927)	0.1896	0.0699	0.0521	0.3271
	MOD M (0.00)	0.3323	0.0531	0.2278	0.4367
	MOD +1 SD (0.69927)	0.4749	0.0767	0.3240	0.6259

n = 308. ***p < 0.001; Inter functional integration; SCA, supply chain agility; CA, competitive advantage; LL, lower limit; CI, confidence Interval; UL, upper limit.

## Discussion

Today's continuously changing business environment creates a big challenge for companies to retain a competitive position in challenging circumstances. Due to continuously changing market trends and customer demands, a firm needs to focus on opportunities as quickly as possible to minimize the risk of business failure or retain their customer. So, for this, valuable information plays a major role in business flourishment. Knowledge management is the firm intangible resource that has a greater impact on firm performance. To identify new opportunities, a firm needs valuable information that helps improve internal processes and operations quickly according to customers' demands. From valuable information, a firm can identify opportunities quickly and minimize the risk of business failure. The valuable information will lead to making better decisions and making changes quickly at the right time. In short, the current study investigates first: the relationship between knowledge management capacities (absorptive, transformative, and inventive) and competitive advantage; second: supply chain agility mediates the relationship between knowledge management capacities and sustainable competitive advantage; third: the moderating role of inter-functional integration between supply chain agility and competitive advantage.

Significantly, knowledge is an intangible asset that enables organizations to achieve superior competitive advantage. In particular, knowledge dominance enhances the firms' capabilities, thereby making the organizations perform to their fullest. It provides an opportunity for the firms to relish the benefit of knowledge management by gaining market-based superiority. However, knowledge management has become a vital tool for fostering business competitiveness. Given the illustration, the study states that today's organizations are increasingly adopting knowledge management practices, thus achieving a competitive advantage (70). Accordingly, due to the increasing significance of the knowledge resource, the organizations are massively embracing novel capabilities, fostering the firms' competitiveness. In this regard, the study shows that absorptive capacity has become a vital construct stimulating the firms' competitiveness (71). Additionally, the research indicates that transformative capacities and innovation capacities also increase the firms' competitiveness. Indeed, our results have supported the prior research findings, thus recording positive results.

Moreover, in today's knowledge economy, supply chain agility plays a role in coping with the increasing market pressures. In explaining this notion, the study states that supply chain agility significantly improves the firms' business performance, ultimately achieving competitive advantage (72). In addition to this, others researchers showed that the knowledge capacities (i.e., AC, TC, and IC) have also accelerated supply chain agility. Similarly, the study also confirmed the mediating role of the supply chain agility to support the previous studies that state that knowledge capabilities (e.g., AC) accelerate the supply chain agility, ultimately gaining competitive advantage (38). In particular, achieving supply chain competitiveness has become a dominant priority of today's firms. In achieving this goal, the study indicates that an organization's inter-functional integration improves the supply chain agility, ultimately the firm's competitiveness (62).

Macro Process suggested by Hayes was used to statistically check the proposed hypotheses H1, H2, H3, H4, and H5 (mediation and moderation analysis). The results revealed that knowledge management capacities (absorptive, transformative, and inventive) are positively and significantly associated with supply chain agility (β = 0.527^***^; β = 0.5093^***^; β = 0.6754^***^) and competitive advantage (β = 0.8672^***^; β = 0.4731^***^; β = 0.6784^***^). Supply chain agility is positively associated with competitive advantage (β = 0.343^***^). Henceforth hypotheses H1, H2, and H3 were approved.

Indirect effects results reveal that supply chain agility fully mediates the relationship between knowledge management capacities (absorptive, transformative, and inventive) and competitive advantage. Whereas, inter-functional integration moderates the relationship between supply chain agility and competitive advantage, the relationship strengthens when inter-functional integration is high.

## Conclusion

Over the past several years, knowledge management has been promoted as an important intangible resource and a necessary factor for an organization to survive in fast-growing business competition; it's vital to achieve competitiveness over rivals and strength in the business circle. Knowledge is an important, valuable asset and has become a serious concern for organizations.

In recent years the mission and vision of business sectors have created novel knowledge and information. The present research is performed to identify the significance of knowledge management capacities and their influence on the competitiveness of the enterprise and the SCA of the firm. Knowledge management capacities (Absorptive, Transformative, and Inventive) have a major influence on the competitive advantage of the firm and supply chain agility. Knowledge management capacities help the firm identify opportunities by acquiring knowledge from an external or internal source or retaining information within a firm. Thus, the more firm has better manage their knowledge management capacities, the more it flourished the work performance internally and will have a significant effect on (SCA).

Knowledge management capacities (KMC) have a significant role in speeding up the supply chain agility to remain competitive in the business circle and target competitors to achieve a competitive position. Thus, the result demonstrates that inter-functional integration has a beneficial impact on (SCA) or has a beneficial influence on the enterprise's competitive position. Inter-functional integration connects employees by sharing and communicating information to accomplish the task quickly and effectively. Inter-functional integration enables the firm to respond quickly to customer need and changing trends by sharing valuable information across the functional units and working together to perform the task effectively or achieve the firm goal.

## Data Availability Statement

The raw data supporting the conclusions of this article will be made available by the authors, without undue reservation.

## Ethics Statement

Ethical review and approval was not required for the study on human participants in accordance with the local legislation and institutional requirements. The patients/participants provided their written informed consent to participate in this study.

## Author Contributions

All authors listed have made a substantial, direct, and intellectual contribution to the work and approved it for publication.

## Funding

This research was supported by the University Research Innovation Fund of Science and Technology Development Center of the Ministry of Education of China (2020ITA05022), the Natural Science Foundation of Hubei Province (2021CFB316), the Preliminary Support Project of Hubei Social Science Foundation (21ZD137), and the Hundreds of Schools Unite with Hundreds of Counties-University Serving Rural Revitalization Science and Technology Support Action Plan (BXLBX0847).

## Conflict of Interest

The authors declare that the research was conducted in the absence of any commercial or financial relationships that could be construed as a potential conflict of interest.

## Publisher's Note

All claims expressed in this article are solely those of the authors and do not necessarily represent those of their affiliated organizations, or those of the publisher, the editors and the reviewers. Any product that may be evaluated in this article, or claim that may be made by its manufacturer, is not guaranteed or endorsed by the publisher.
